# Characterization of social behavior in young and middle-aged ChAT-IRES-Cre mouse

**DOI:** 10.1371/journal.pone.0272141

**Published:** 2022-08-04

**Authors:** Cyril Lhopitallier, Charlotte Perrault, Frédéric Chauveau, Françoise Saurini, Sylvie Berrard, Sylvie Granon, Alexis Faure

**Affiliations:** 1 Neurobiology of Decision Making, Paris-Saclay Institute of Neuroscience (NeuroPSI), CNRS, University Paris-Saclay, Gif-sur-Yvett, France; 2 University of Turku, Turku, Finland; 3 Institut de Recherche Biomédicale des Armées (IRBA), Brétigny sur Orge Cedex, France; 4 Université de Paris, NeuroDiderot, Inserm, Paris, France; University of Illinois at Urbana-Champaign, UNITED STATES

## Abstract

The cholinergic system is an important modulator of brain processes. It contributes to the regulation of several cognitive functions and emotional states, hence altering behaviors. Previous works showed that cholinergic (nicotinic) receptors of the prefrontal cortex are needed for adapted social behaviors. However, these data were obtained in mutant mice that also present alterations of several neurotransmitter systems, in addition to the cholinergic system. ChAT-IRES-Cre mice, that express the Cre recombinase specifically in cholinergic neurons, are useful tools to investigate the role of the cholinergic circuits in behavior. However, their own behavioral phenotype has not yet been fully characterized, in particular social behavior. In addition, the consequences of aging on the cholinergic system of ChAT-IRES-Cre mice has never been studied, despite the fact that aging is known to compromise the cholinergic system efficiency. The aim of the current study was thus to characterize the social phenotype of ChAT-IRES-Cre mice both at young (2–3 months) and middle (10–11 months) ages. Our results reveal an alteration of the cholinergic system, evidenced by a decrease of *ChAT*, *CHT* and *VAChT* gene expression in the striatum of the mice, that was accompanied by mild social disturbances and a tendency towards anxiety. Aging decreased social dominance, without being amplified by the cholinergic alterations. Altogether, this study shows that ChAT-IRES-Cre mice are useful models for studying the cholinergic system‘s role in social behavior using appropriate modulating technics (optogenetic or DREADD).

## Introduction

The cholinergic system is an important modulator of brain functions [1]. Most cholinergic projecting neurons originate from two brain regions, the basal forebrain and the brainstem [2]. Basal forebrain cholinergic neurons, whose cell bodies lie in the medial septum, the nuclei of diagonal band of Broca, the nucleus basalis of Meynert, and the substantia innominata, innervate almost every brain areas, including the prefrontal cortex (PFC), the amygdala and the hippocampus [2–5]. In the brainstem, cholinergic neurons from the laterodorsal and pedunculopontine tegmental nuclei (LDTg/PPTg) project axons to subcortical regions, among which the substantia nigra (SN), the ventral tegmental area (VTA), the thalamus, the hypothalamus, the amygdala, and the striatum, as well as to pontine targets [6,7]. In addition, local cholinergic interneurons are present in the cortex and in the striatum. While the major cholinergic innervation of the cortex originates from the basal forebrain, the striatal cholinergic interneurons, despite their low number (1–3% of striatal neurons), densely innervate the entire striatum and constitute the principal source of acetylcholine (Ach) in this structure [8–10].

Given the extensive cholinergic innervation of the brain, cholinergic receptor regulate the activity of different neurotransmitter systems and contribute to the modulation of cognitive functions, emotional states and behaviors, such as attention [4,11], learning and memory processes [12], decision making [13–17], or behavioral flexibility [1,5].

Social cognition, that describes all processes involved in the ability to establish adapted social interactions [18,19], recapitulates numerous cognitive functions that could be modulated by the cholinergic system, as developed above, and that rely on the mobilization of fronto-striatal circuits [13,18–20]. During social interactions, rodents emit ultrasonic vocalization (USVs) which frequency level was shown to be related to the emotional status of the animal [21–24]. Our previous results using mice lacking the β2 subunit of nicotinic cholinergic receptors (β2KO), pointed to the involvement of acetylcholine (ACh) in social behavior through its necessary and sufficient action on neuronal nicotinic receptors (nAChRs) in the PFC [13,25]. Moreover, selective lesion of striatal cholinergic interneurons decreased social motivation [26] and impaired selection of action [8,27,28], suggesting their important role in social interaction.

However, the use of β2KO mice to question the specific involvement of the cholinergic system in social interaction is limited by the fact that these mice display dysregulation of multiple neurotransmission systems, particularly in the PFC and the hippocampus [29]. This hampers the characterization of the precise role of ACh during social behavior in this mouse model. One way to address this question is to directly modulate the release of ACh in target areas during social interaction tasks. Controlling specifically the activity of cholinergic neurons can be achieved using optogenetic or chemogenetic (DREADD) approaches [4,30–33]. These methods rely on the cell-specific expression (Cre-inducible) of a light-sensitive protein, such as opsin or channelrhodopsin-2 (ChR2), or of modified receptors, in genetically modified mice expressing the Cre recombinase exclusively in cell types of interest. In the case of cholinergic neurons, ChAT(BAC)Cre transgenic mice and the ChAT-IRES-Cre mouse line have been frequently used. In both types of mouse models, the expression of Cre recombinase is driven by the promoters and the regulatory elements of the gene encoding choline acetyltransferase (ChAT), the biosynthesis enzyme of ACh. This gene has the unique property to contain in its first intron the gene of another key cholinergic protein, namely the vesicular acetylcholine transporter (VAChT), which translocates cytoplasmic ACh into synaptic vesicles. These two genes form the cholinergic gene locus [34–37]. The third protein required for the cholinergic presynaptic function, whose gene lies on a different chromosome, is the high-affinity choline transporter (CHT), that imports extracellular choline used for ACh synthesis into cholinergic nerve terminals [38–40].

Most of the BAC clones used to generate BAC transgenic lines contain large genomic fragments encompassing both the gene of interest and unwanted adjacent genes. These extra genes are introduced in the genome in multiple copies with the BAC transgene, and their unintended overexpression may have functional consequences [41]. This is particularly the case of ChAT-BAC constructs, in which the ChAT gene is disrupted by the transgene, but that contain the active VAChT gene. Since VAChT controls cholinergic signaling [42,43], its overepression in transgenic animals may, depending on the copy number, lead to an increase of the cholinergic tone associated to behavioral alterations, as reported for ChAT-ChR2-EYFP mice or ChAT-Cre transgenic [44,45]. In contrast, ChAT-IRES-Cre mice were created by a knock-in strategy, by inserting an internal ribosome entry site (IRES) fused to a Cre allele downstream of the stop codon of the *ChAT* gene [46]. Cre recombinase is thus expressed under control of the endogenous *ChAT* gene promoters, and the *VAChT* gene is theorically not overexpressed in these mice. This was actually confirmed in the hippocampus, where the level of the VAChT protein is equivalent to that of WT controls [47]. In contrast, in the hippocampus of ChAT-BAC-Cre mice, the amount of VAChT is greater than in WT animals [47]. Moreover, the Cre recombinase is expressed at higher levels in ChAT-BAC-Cre mice than in ChAT-IRES-Cre mice [47], which should not be overlooked, because Cre might be toxic for cholinergic neurons [48–51]. Together, all the above data strongly support the use of ChAT-IRES-Cre mice for targeting cholinergic neurons in order to dissect the relative contribution of cholinergic circuits to mouse behavior by optogenetic or chemogenetic approaches. However, it is first necessary to thoroughly characterize their basic behavioral phenotype. It was previously shown that ChAT-IRES-Cre mice display normal anxiety level, drug-induced cataplexy or nicotine self-administration behavior relative to WT littermates. Nevertheless, they exhibit a significant hypo-locomotor phenotype and a delayed acquisition of an operant food conditioning [47]. Yet, this mouse line is far from being fully described in terms of cognitive phenotype, particularly regarding social abilities. It is thus important to explore in depth the social phenotype of this mouse line to validate and interprete data from social cognition experiments.

Another important issue that needs to be addressed is the age-effect of the cholinergic system in the ChAT-IRES-Cre mice. Indeed, a cholinergic hypothesis postulates that age-related cognitive alterations during normal or pathological aging could be sustained by cholinergic dysfunction with age [52–58]. Among age-related alterations in cognition [59–61], social cognition was disrupted with a loss of social interaction from young adult (3 months) to aged rat (30 months old) [62,63] or from young mice to middle-aged mice [63]. Accordingly, in mice, there is evidence of an age-related decrease of social interactions [62]. More precisely, in an extensive comparison of the behavioural phenotype of mice at different ages [2–3;4–5;6–7;8–12 months], social deficit begins as early as 11-12-months [63]. The arising question is to know whether ChAT-IRES-Cre mice, that have moderate cholinergic system alteration, show earlier evolution of age-related cognitive impairments.

The aim of this study was to determine the social phenotype of ChAT-IRES-Cre in young adult mice and in middle-aged mice. For this purpose, we used a social interaction task that allows free social interaction altogether with vocal communication. We measured also social motivation, sensory abilities and anxiety levels. Then, we quantified by RT-qPCR the expression of genes involved in the biosynthesis and vesicular transport of ACh in the dorsal striatum, that contains cholinergic interneurons. We have not targeted cholinergic neurons projecting to the prefrontal cortex as we acknowledged that dissection of the nucleus basalis of Meynert was non-sufficiently reproducible. We thus chose to target the cholinergic interneurons of the dorsal striatum as a proxy for the cholinergic system function and because of its implication in social motivation.

## Materials and methods

### Animals

ChAT-IRES-Cre mice, hereby called ChAT-Cre mice, were originally obtained from The Jackson Laboratory (B6; 129S6Chattm2(cre)Lowl/Lowl/J; https://www.jax.org/strain/006410; RRID:IMSR_JAX:006410). Initially backcrossed with C57BL6 mice obtained from the Charles River’s laboratory and maintained by Heterozygous X Heterozygous mating, the ChAT-Cre mice are now bred as homozygous-homozygous mating pairs in our animal facility. C57BL/6J mice were either obtained from Charles Rivers Laboratories France (L’Arbresle Cedex, France) for middle-aged ones or bred in our animal facility for young ones. They are hereby named “WT”.

All experiments were conducted in accordance with the local regulations for animal experiments as well as the recommendations issued by EU Directive 2010/63/EU, Decree N 2013–118 of February 1, 2013, and the French National Committee (87/848). Ethical project reference was 2014–13.

Male mice arrived at the animal facility at eight weeks of age (obtained from Charles Rivers) or after weaning (home bred), and were maintained in collective cages (3 to 4 mice per cage). Animals were maintained in 20.5 * 32.5 cm home cages with water and food *ad libitum* and a circadian light cycle of 12h light /12h dark (beginning at 8 am). Young mice were 2–3 months old (n = 8 ChAT-Cre and 8 WT mice) and middle-aged mice were 11–12 months old (n = 7 ChAT-Cre mice and 8 WT mice). On the figures, the animals were noted WT young, ChAT-Cre young; WT M.A. (Middle age); ChAT-Cre M.A. (middle age).

All mice tested were male and housed individually 3–4 weeks before social experiments. Mice used as visitor animals for social experiments (young n = 6, and middle-aged n = 6, WT mice) were maintained in social cages (see protocols).

**Fig 1:**
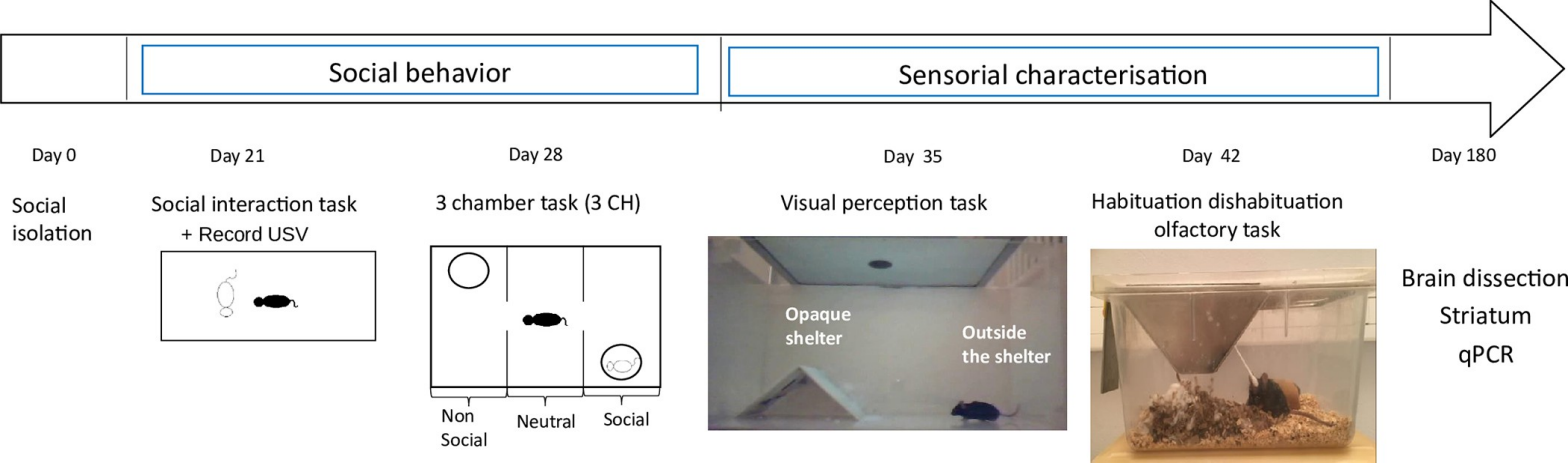
Timeline of experiments. In the social interaction and in the three-chamber tasks, the black mouse represents the Host mouse and the white mouse represents the Visitor mouse.

Fig 1 shows the timeline of experiments. The mice were two months old before beginning social isolation. After three weeks of isolation, the social interaction task was carried out, then one week after, the three-chamber task which controls for social motivation was performed. To check for sensory deficit, a visual perception task was carried out 35 days after the beginning of the experiment. Finally, one week after the visual experiment, an olfactory test was conducted. After the handling experiment, we quantified by RT-qPCR the expression of genes involved in the biosynthesis and vesicular transport of ACh in the dorsal striatum, that contains cholinergic interneurons.

#### The 3 chambers (3 CH)

The apparatus was a rectangular Plexiglas box 64*42 cm divided in three compartments of equal surface. On the two opposite compartments, we placed two Plexiglas cups (diameter 9.2cm; H 10.2 cm) with 30 holes. Diffuse light was set at 100 lux. An animal (host mouse) was placed at the center of the apparatus and could freely explore the environment for 15 min. After this habituation period, another animal (visitor mice, unfamiliar, same sex and age) was placed under a Plexiglas cup. The visitor mouse cannot move freely, but it can poke with its nose into holes and touch the host mouse. The chamber with the visitor mouse is referred to as “social chamber”, while the empty chamber is referred to as “non-social chamber”. Neutral chamber is the one in between (Fig 1). Localisation of the visitor mouse was counterbalanced between each experiment. The behavior of the host mouse was recorded during 8 min by a webcam connected at the ANY-maze software. The behavioral parameters analysed off-line were the following: total distance traveled, total immobility duration, maximum speed, time spent, immobility duration and distance traveled in each chamber, time spent in contact with the congener.

The time spent in contact with the congener was evaluated when the host mouse pokes with its nose into holes and touches the visitor mouse.

#### Social interaction task

The Social Interaction Task (SIT, see Fig 1) was previously described in detail [64,65]. Briefly, the isolated mouse (i.e., “host mouse”) was placed in a transparent Plexiglas cage (L 50 cm * l 25 cm * h 30 cm) in an unfamiliar quiet room for 30 min of habituation. The diffuse light in the room was set at 100 Lux. After the habituation session, a “visitor mouse” was introduced into the cage. The two animals can establish spontaneous, free and reciprocal social interactions that were recorded for 8 min by a numeric video camera (webcam) connected to a computer. The mouse behavior was analysed off-line with the Solomon Coder software (version beta 19.08.02) developed by A. Peter. We distinguished multiples categories within social behaviors:

- Affiliative behaviors: number and duration of contacts initiated by host or visitor mice, i.e. when animals touched each other with various parts of the body.
- Dominance: number and duration of host or visitor follow episodes. Other parameters of dominance were scored, such as “paw control” that refers to the number of times the host mouse placed its forepaws on the head or the back of the visitor.
- Escaping: the number and duration of escape behaviors were scored every time the host or visitor mouse interrupted a contact.
- The number of rearing was scored as a marker of exploratory behavior.
- Aggressive behavior was reflected by the number of bites and latency to attack.

#### USV recordings

USVs were recorded during the 8 min of the social interaction task. A condenser ultrasound microphone Polaroid/CMPA was placed above the experimental chamber, high enough so that the receiving angle of the microphone covered the whole area of the test. It was connected to an ultrasound recording interface Ultrasound Gate 416H, which was itself plugged into a personal computer equipped with the recording software Avisoft Recorder USG (Sampling frequency: 250 kHz; FFT-length: 1024 points; 16-bits). All recording hardware and software were from Avisoft Bioacoustics H (Berlin, Germany). USVs were analysed off-line with SASLab Pro (Avisoft Bioacoustic H, Berlin, Germany). Spectrograms were generated for each detected call (Sampling frequency: 250 kHz; FFT-length: 1024 points; 16-bit; Blackman window; overlap: 87.5%; time resolution: 0.512 ms; frequency resolution: 244 Hz). We categorized the waveform pattern of each call as belonging to one of ten distinct categories based on their duration and frequency modulation during all the social interaction task: Short S, Flat F, one frequency jump OFJ, Multiple frequency jumps MFJ, U-shape U, Chevron C, Modulated M, Composite W, Upward H, Downward D and an undefined UND [22]. Calculations of the percentages of the repertoire considered only the animals of each group that emitted calls. USVs of one middle-aged ChAT-Cre mouse and of one young WT mouse were discarded due to technical problems.

#### Visual perception test: Defensive response to a threatening visual stimulus

This protocol was described by [66]. The aim of this test is to assess the mouse visual ability using ethological defensive reactions toward visual stimuli mimicking a predator that appears from the ceiling. The apparatus consists in a Plexiglas cage (L 50 cm * l 25 cm * h 30 cm) covered by a computer screen and containing a cardboard shelter. The animal was habituated in this environment for 10 min, after which the “looming stimulus” was on. On a grey screen a black disc appears directly above the animal at a diameter of 2 degrees of visual angle, expanded to 20 degrees in 250 ms, and remained at that size for 250 ms. This stimulus was repeated 15 times with 500 ms pauses. The looming stimulus lasted 15 s. The habituation and the reaction to the stimulus were recorded with a camera (Logitech) placed at 40 cm in front of the cage. During all the experiment, the diffuse light was set at 100 lux.

We measured off-line the latency of the first reaction during the looming stimulus, and also the percentage of freezzing during the threatening stimulus.

All mice already under the opaque shelter when the looming stimulus started were excluded from the analysis because we couldn’t be sure that they perceived the stimulus and therefore we couldn’t precisely determine the latency of their reaction. Three middle-aged ChAT-Cre and one young WT mice were excluded for that reason (see Fig 1 for experimental set up).

#### Habituation/Dishabituation olfactory test

The protocol is adapted from [67]. This task allows to test whether mice are able to detect and discriminate different odors, among which social and non-social odors. Three odors were presented in this test: a neutral (water), a non-social (almond, Vahiné®) diluted at 1/100 in distilled water, and a social odor. Social odor was obtained from two cages containing mice of the same sex by swabing cottons at the bottom the cages. All odors were presented on cotton swabs and were prepared fresh daily.

Mice were tested in their homecage in an unfamiliar experimental room. A clean cotton swab was placed for 30 min through the grid of the cage so that the animal could sniff it. This step allows habituation to this new object. Next, odors were presented sequentially on a novel cotton swab in the following order: water, almond, social odor. Each odor was presented 3 times during 2 min trials with 1 min inter-trial intervals. The sniffing duration was recorded for each odor (see Fig 1).

The habituation was measurement by comparing the results from the three exposure to the same odor. If there was a significative difference, we considered that the mouse habituated to the odor.

The dishabituation score consisted in a significant difference between the third odor presentation and the first presentation of the new odor.

#### Reverse transcription and real-time quantitative PCR analysis

Total RNA was purified from dorsal striatum using RNeasy micro kit RNA extraction columns according to the manufacturer’s instructions (Macherey-Nagel, France) after tissue homogenization in NucleoZOL^TM^. RNA concentrations were quantified by spectrophotometry (Nanodrop^TM^, Thermofisher Scientific, MA, USA). Reverse transcription (RT) was performed from 500 ng of total RNA using the iScriptTM cDNA synthesis kit (Bio-Rad, Marnes-la-Coquette, France) for 40 min at 42°C. Real-time quantitative PCR (qPCR) was conducted in a CFX384 Real Time PCR detection System (Biorad, Hercules, CA, USA). qPCR reactions were performed in triplicate from 5 ng cDNA using the SYBR Green Super-mix (Bio-Rad) in a total volume of 10 μl, in the following 2-step cycling condition: initial denaturation (30 s at 98°C) and 45 cycles of 8 s at 95°C (denaturation) and 20 s at 60°C (annealing and extension). Negative controls (water or RT controls carried out without reverse transcriptase instead of cDNA) were included in each PCR run. Amplification specificity was assessed by melting curve analysis. The following pairs of primers were designed using Primer3 plus or Primer blast softwares and were chosen on different exons, except for VAChT, which does not contain introns in mammals [34,36]: 5’-CCAGCTAAGGTTTGCAGCCA-3’ (forward) and 5’-CCAGTCAGTGGGAATGGATTG-3’ (reverse) for *ChAT* (85 bp); 5’-CTTTTGCCCTGTCACATCCT-3’ (forward) and 5’-TCCACCCAGCATCAATAACA-3’ (reverse) for *CHT* (143 bp), 5’-TCATGTTCGCCTCCACAGTC-3’ (forward) and 5’- AGGCTCCTCGGGATACTTGT-3’ (reverse) for *VAChT* (142 bp) and 5’-ACAGCCACTCTGGAGGAGAA-3’ (forward) and 5’-GAGTCCGTTGGTCTTGAGGA-3’ (reverse) for *Rpl13a* (146 pb).The efficiency of each pair of primers was between 95 and 105%. The expression of each gene of interest was normalized to that of the housekeeping gene *Rpl13a* assessed in the same PCR run, and was calculated using the comparative Ct method (2^−ΔΔC^_T_). Data are presented as relative transcript mRNA abundance with respect to young WT mice.

### Statistical analysis

Non-parametric analyses were performed using R software (version 3.5.0 of the R foundation for Statistical computing with Rcmdr-package) as part of behavioral data that were not following a normal distribution. We used the Kruskal-Wallis chi squared test to assess for global difference between groups (WT young; ChAT-Cre Young, WT M.A and ChAT-Cre M.A.). This statistic is refered to as group effect (noted H in the Result section). We use Wilcoxon rank sum test (noted W in the Result session) when comparing two independent groups and paired Wilcoxon rank sum test (noted V in the Result session) when comparing two behavioral measures of the same animals. Gene expression data were analysed using analysis of variance (ANOVA) with genotype and age as factors (Graphpad Prism 8 software, San Diego, CA). Significant main effects were further analyzed by Bonferroni *post-hoc* comparisons of means. Statistical significance was set at p < 0.05. Principal component analysis (PCA) was performed using the FactomineR package. The missing data were replaced by the mean of the group for this parameter.

## 3) Results

### 3.1) Organisation of social behavior in the Social interaction task (SIT)

**Fig 2:**
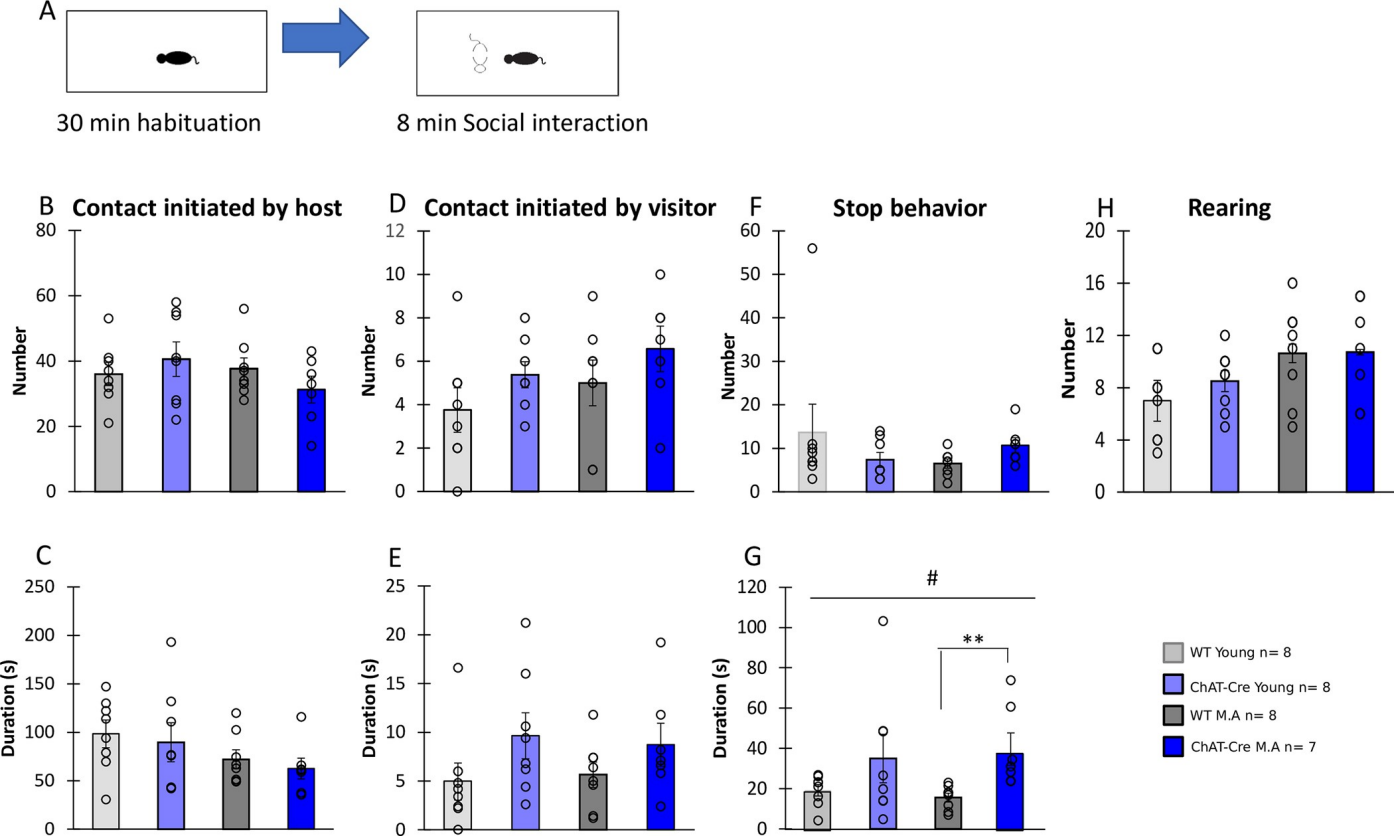
Contact; stop and exploration in the Social Interaction Task. **A)** Schematic representation of the social interaction task. B) Number of contacts initiated by Host mice. C) Duration of contact initiated by Host mice. D) Number of contacts initiated by Visitor mice. E) Duration of contact initiated by Visitor mice. F) Number of stops episodes. G) Duration of stop episodes. H) Number of rearings made by Host mice. Host mice were previously isolated for 3 weeks and exposed to the novel experimental environment for 30 minutes before being presented with a visitor mouse: WT young n = 8, ChAT-Cre young n = 8, WT Middle Age n = 8, ChAT-Cre Middle Age n = 7. Visitor mice (young n = 6 and middle age n = 6) were WTs of same age and sex maintained in a social cage before the experiment. Data are presented as means ± SE; *p < 0.05; **p<0.01; ***p<0.001; Kruskal-Wallis rank-sum test analysis then Wilcoxon and d signed-rank tests were applied when appropriate. # represents the genotype effect and ◆ the age effect. Kruskal-Wallis results were not represented for simplicity.

This test allows evaluation of free social interation. A set up of experiment is shown in Fig 2A. During the social interaction task (SIT), ChAT-Cre mice exhibited normal affiliative behaviors that were not affected by age. All groups showed an equivalent number of contacts and duration of contacts initiated by host animals (H = 5.11, df = 3, NS,H = 4.67, df = 3, NS, Fig 2B and 2C, respectively) or by visitor animals (H = 1.66, df = 3, NS, H = 5.86, df = 3, NS respectively, Fig 2D and 2E).

We previously showed that stop behaviors in the SIT are especially important as they are choice points opening towards multiple possible behavioral outputs [64,65]. Age or genotype had no significant impact on the number of stops (H = 4.46, df = 3, NS, Fig 2F). However, groups significantly differed in stop duration (H = 9.50, df = 3, p<0.05, Fig 2G), with ChAT-Cre mice making significantly longer stops than WT animals (genotype effect W = 185, p<0.05). There was no impact of age (age effect; W = 115, NS, Fig 2G). However, this genotype effect was significant only in middle-aged animals (ChAT-Cre young vs WT young: W = 39, NS, ChAT-Cre M.A vs WT M.A: W = 54, p <0.01, Fig 2G), likely because of an important variability within the ChAT-Cre young group. Genotype or age didn’t significantly alter the rearing exploratory behavior (H = 5.73, df = 3, NS, Fig 2H).

**Fig 3:**
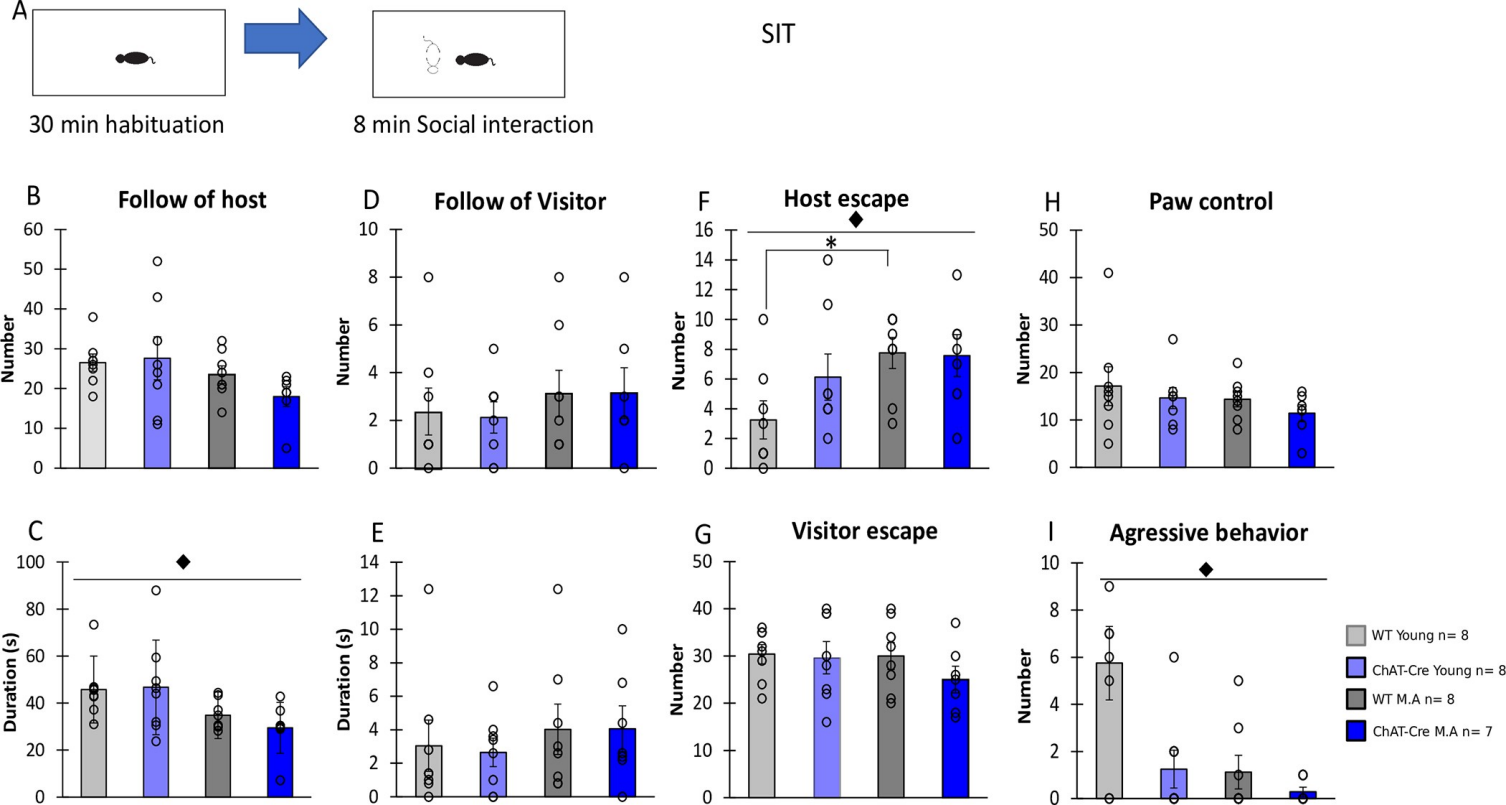
Behavior in the Social Interaction Task. **A)** Schematic representation of the social interaction task procedure. B) Number of follow episodes made by Host mice. C) Duration of follow episodes (i.e., following while making oro-genital contact) made by Host mice. D) Number of follow episodes made by Visitor mice. E) Duration of follow episodes made by Visitor mice. F) Number of escape (i.e., interuption of contact) episodes made by Host mice. G) Number of escape episodes made by Visitor mice H) Number of paw control made by Host mice (i.e., number of times Host mice put their forepaws on the head or back of Visitor mice). I) Number of aggressive episodes of Host mice. WT young n = 8, ChAT-Cre young n = 8, WT Middle Age n = 8, ChAT-Cre Middle Age n = 7. Visitor mice (young n = 6 and middle age n = 6). Data are presented as means ± SE; *p < 0.05; **p<0.01; ***p<0.001; Kruskal-Wallis rank-sum test analysis then Wilcoxon and d signed-rank tests were applied when appropriate. # represents the genotype effect and ◆ the age effect. Kruskal-Wallis results were not represented for simplicity.

In contrast, even if the number of following behaviors of the host animal was equivalent in all groups (H = 6.23, df = 3, NS, Fig 3B), durations of these events significantly differed between groups (H = 10.60, df = 3, p<0.05, Fig 3C). In comparison with young animals, middle-aged mice showed a decrease of following behavior duration, independently of the genotype (age effect, W = 199, p <0,01; genotype effect, W = 101.5, NS, Fig 3C). The number and duration of following behavior exhibited by the visitor mice were comparable in all groups (H = 0.93, df = 3, NS, H = 1.08, df = 3, NS respectively, Fig 3D and 3E).

Interestingly, dominance duration decreased with age and paralleled the increase of the number of escapes from the host animal. Although host mice of all groups exhibited identical number of escapes (H = 7.33, df = 3, NS), middle-aged animals displayed a greater number of escapes than young animals (age effect: W = 64.5, p<0.05) without any genotype effect (W = 143, NS, Fig 3F). This age effect on the number of escapes from the host was restricted to WT animals (age effect in WT: W = 10.5, p<0,05; age effect in ChAT-Cre: W = 19.5, NS, Fig 3F). Escape behavior made by visitor mice didn’t differ between groups (H = 2.40, df = 3, NS, Fig 3G).

There was no significant difference for the number of “paw control” between groups (H = 2.34, df = 3, NS, Fig 3H).

The decreased dominance displayed by middle-aged mice was also observed for aggressive behavior (Fig 3I): There were no significant group or genotype effects (group: H = 8.87, df = 3, NS, genotype: W = 77.5, NS), but a significant effect of age (W = 165.5, p = 0.051, Fig 3I). This difference can be explained by a significant reduction of aggressive behavior only in WT animals (age effect in WT: W = 52.5, p <0.05; age effect in ChAT-Cre: W = 33.5, NS).

**Fig 4:**
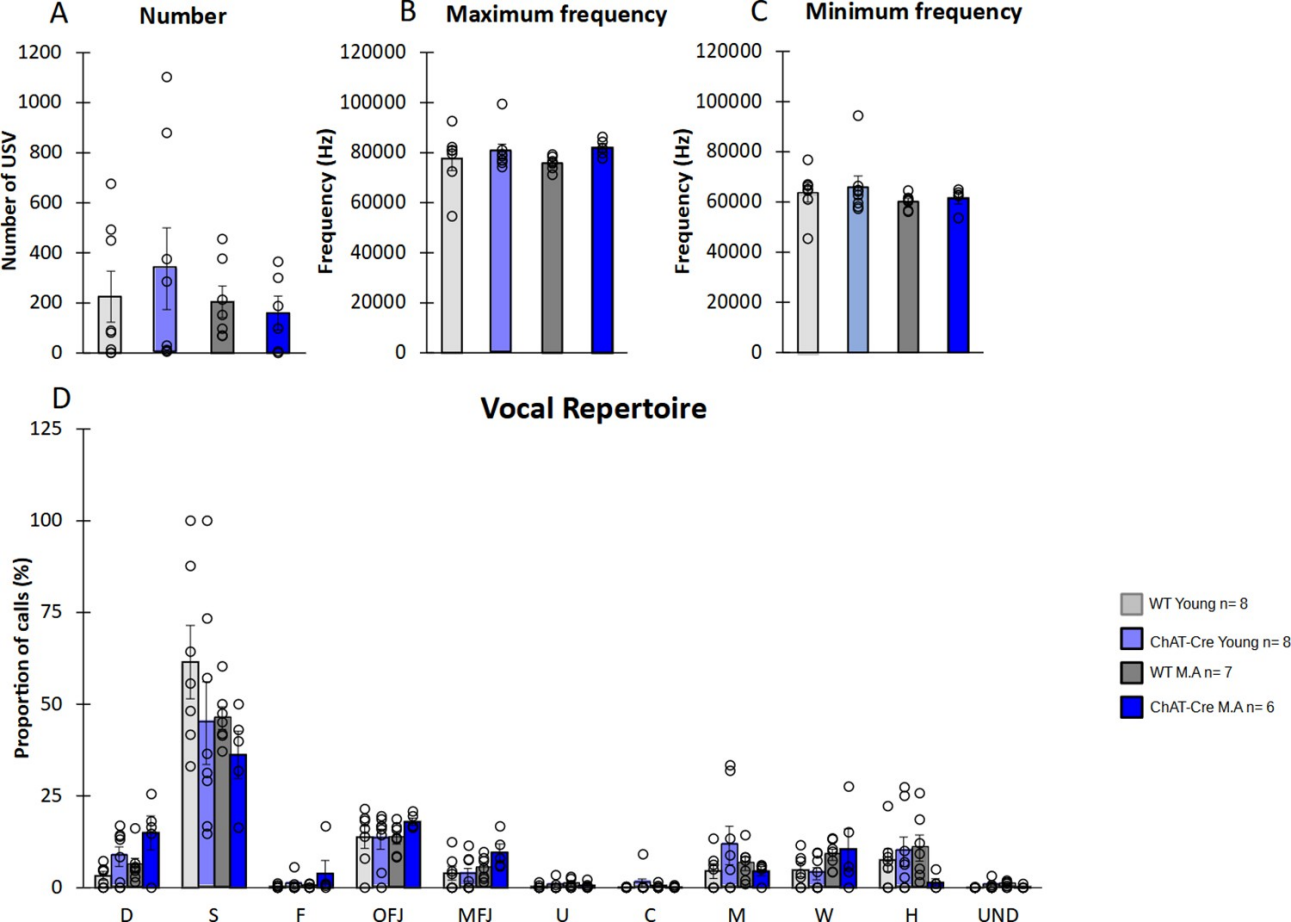
Ultrasonic vocalizations (USVs) emitted during SIT. **A)** Number of USV. B) Maximum frequency of USVs. C) Minimum frequency of USVs. D) Diversity of USVs recorded. WT young n = 8, ChAT-Cre young n = 8, WT Middle Age n = 8, ChAT-Cre Middle Age n = 7. Visitor mice (young n = 6 and middle age n = 6). Data are presented as means ± SE; *p < 0.05; **p<0.01; ***p<0.001; Kruskal-Wallis rank-sum test analysis then Wilcoxon and d signed-rank tests were applied when appropriate. # represents the genotype effect and ◆ the age effect. Kruskal-Wallis results were not represented for simplicity.

Ultrasonic vocalisations (USVs) were recorded during the SIT. USVs are emotional markers. All groups didn’t show any significant difference for neither the number of USVs (H = 0.1034, df = 3, NS, Fig 4A), nor their frequencies (maximum frequency H = 7.32, df = 3, NS, Fig 4B, Minimum frequency H = 4.22, df = 3, NS, Fig 4B and 4C). When we looked at the vocal repertoire (i.e., the percentage of calls uttered by animals), no significant difference was found between groups (C: H = 2.77, df = 5, NS; D: H = 7.78, df = 5, NS; F: H = 4.5, df = 5, NS; H: H = 5.99, df = 5, NS; M: H = 2.27, df = 5, NS; MFJ: H = 6.7881, df = 5, NS; OFJ: H = 6.68, df = 5, NS; S: H = 6.18, df = 5, NS; U: H = 4.03, df = 5, NS; UND:H = 9.96, df = 5, NS; W: H = 9.04, df = 5, NS, Fig 4D). Age and genotype thus did not affect the vocal repertoire of mice.

Taken together, these results showed that the ChAT-Cre mice differed from WTs only for stop behavior. Moreover, dominant behaviors and aggressivity decreased with age.

### 3.2) Social motivation in the three-chamber task

**Fig 5:**
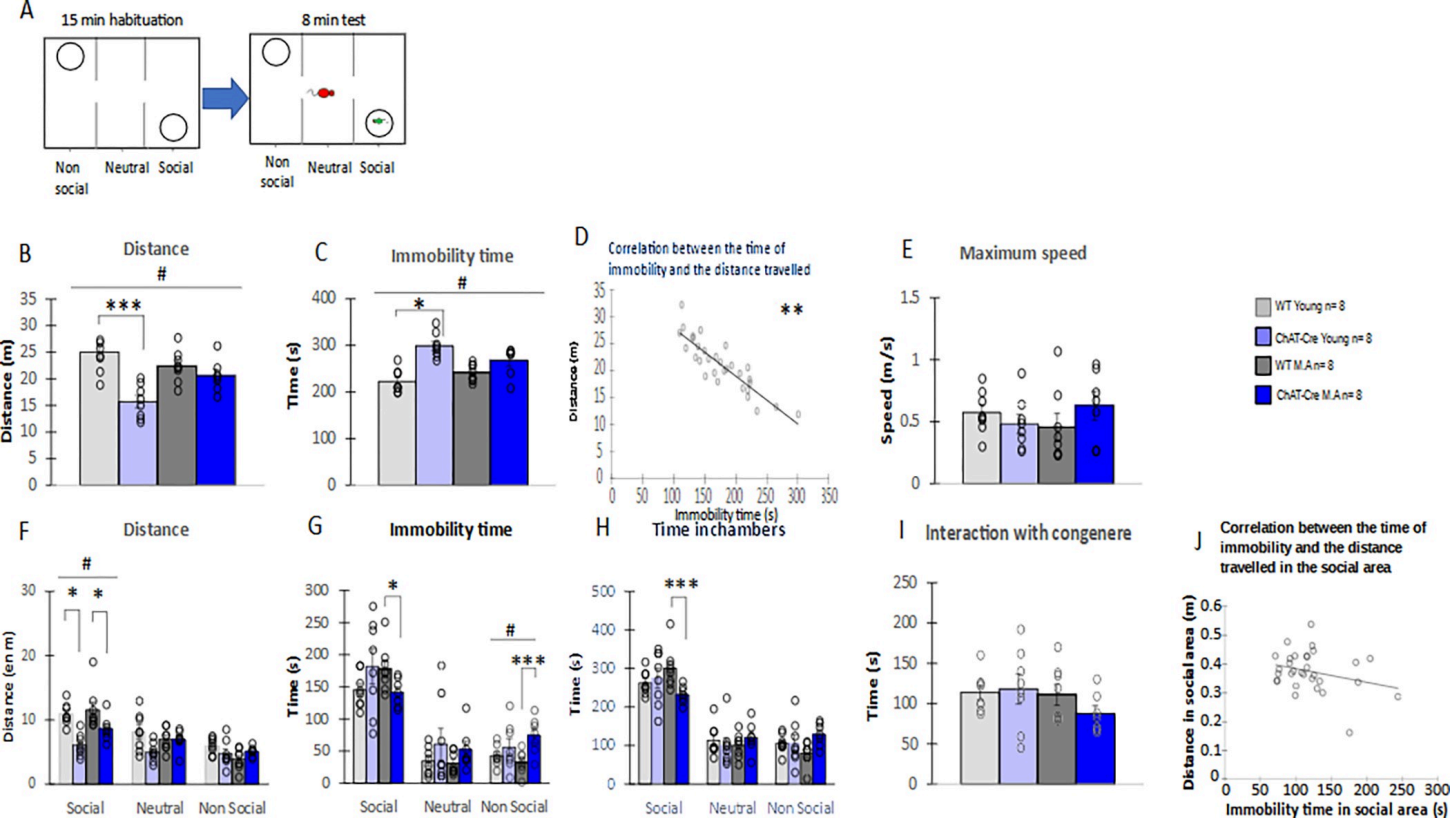
Behavior in the 3-chamber task. **A)** Procedure. Host mice used for this experiment were WT Young n = 8; ChAT-Cre Young n = 8; WT Middle Age (M.A) n = 8; ChAT-Cre Middle Age (M.A) n = 7. The visitor mice used were all WTs of same age and sex than host mice (young n = 6, M.A. n = 6) maintained in social cages. B) Distance travelled. C) Immobility time. D) Correlation between the time of immobility and the distance travelled. E) Maximum speed. F) Distance travelled in chambers. G) Immobility time in chambers. H) Time spent in chambers I) Time of interaction with the congener. J) Correlation between the time of immobility and the distance travelled in the social area. Data are presented as means ± SE; *p < 0.05; **p<0.01; ***p<0.001; Kruskal-Wallis rank-sum test analysis then Wilcoxon and d signed-rank tests were applied when appropriate. # represents the genotype effect and υ the age effect. Kruskal-Wallis results were not represented for simplicity.

The three-chamber task test allows evaluation of social motivation in mice. A set up of experiment is shown in Fig 5A. In this test, ChAT-Cre mice travelled significantly less distance than WT animals (group effect: H = 16.63, df = 3, p <0.001, genotype effect: W = 32, p <0.001), and there was no significant effect of age (age effect: W = 99, NS, Fig 5B). Moreover, the ChAT-Cre mice were significantly more immobile than WT animals (H = 18.304, df = 3, p <0.001, genotype effect: W = 219, p<0.001) and age had no effect on immobility duration (W = 131, all NS, Fig 5C). There was a negative correlation between the travelled distance and the time of immobility (S = 7690, p <0.01, Fig 5D), and groups didn’t differ in terms of maximal speed (H = 3.81, df = 3, NS, Fig 5E), suggesting that the lower distance covered by the ChAT-Cre mice relative to WT controls cannot be explained by a global motor alteration, but rather by an increase of immobility time. Only ChAT-Cre young animals showed a significant reduction of distance travelled along with increased immobility compared to WT young (ChAT-Cre young vs WT young effect for distance W = 2, p<0.001 and for immobility time W = 63.5, p<0.01), by comparison with middle-aged mice (ChAT-Cre M.A vs WT M.A for distance W = 17, NS and for time of immobilily W = 44, NS, Fig 5B and 5C).

In order to evaluate social motivation, we distinguished each measure taken in the 3 chambers. Results showed a normal social motivation in all groups, with significantly greater distance travelled (V = 480, p <0.001, Fig 5F) and time of immobility (V = 496, p <0.001, Fig 5G), time spent (V = 495, p <0.001, Fig 5H), in the social chamber by comparison with the non-social one (Fig 5F–5H). Moreover, all groups displayed an equivalent time of interaction with the congener, i.e. duration of nose-nose contact through holes in the plexiglas cup (H = 3.43, df = 3, NS, Fig 5I).

In the neutral chamber, there was no significant difference between groups regarding the distance travelled, the time of immobility or the time spent (H = 7.65, df = 3, NS; H = 2.02, df = 3, 0.055; H = 2.76, df = 3, NS, respectively, Fig 5F–5H).

In the non-social chamber, groups differed significantly for time of immobility (H = 7.93, df = 3, p <0.05, Fig 5G) but not for time spent or distance travelled (H = 6.79, df = 3, NS; H = 7.07, df = 3, NS respectively, Fig 5F and 5G). ChAT-Cre mice were significantly more immobile than WT animals (genotype effect: W = 180, p <0,01) and this genotype effect was restricted to middle-aged animals (ChAT-Cre young vs WT young: W = 38, NS; ChAT-Cre M.A vs WT M.A middle-aged W = 54, p < 0.001, Fig 5H). Age had no significant impact on the immobility parameter (W = 118, NS).

In the social chamber, almost behavioral measures significantly differed between groups (distance travelled: H = 19.136, df = 3, p <0.001, time spent: H = 8.26, df = 3, p <0.05, Fig 5F–5H) but not the immobility time (H = 5.60, df = 3, NS). More precisely, ChAT-Cre mice traveled significantly less distance in the social chamber than WT animals, with no evolution with age (genotype effect: W = 17, p <0.001; age effect: W = 90, NS, Fig 5F). Regarding the immobility time and the time spent in the social area, global group effect was neither due to a genotype effect (W = 112, NS; W = 81, NS), nor to an age effect (W = 117, NS; W = 128, NS, Figs 2H and 5F). However, a more careful examination showed that this global effect might be due to a significant decrease of time of immobility and time spent displayed specifically by middle-aged ChAT-Cre mice as compared to WT mice (middle-aged: time of immobility, W = 9, p < 0.05 and time, W = 3, p <0.001; NS; Young: Time of immobility, W = 43, NS, and time, W = 37, NS). Moreover, there was no correlation between the distance travelled and the time of immobility in the social chamber (S = 4608, NS, Fig 5J).

These data showed that ChAT-Cre young mice exhibit normal social motivation, which is maintained even at middle-aged. However, ChAT-Cre mice showed a global reduction of the distance travelled related to a greater immobility in this test.

### 3.3) Reaction to a visual threatening stimulus

**Fig 6:**
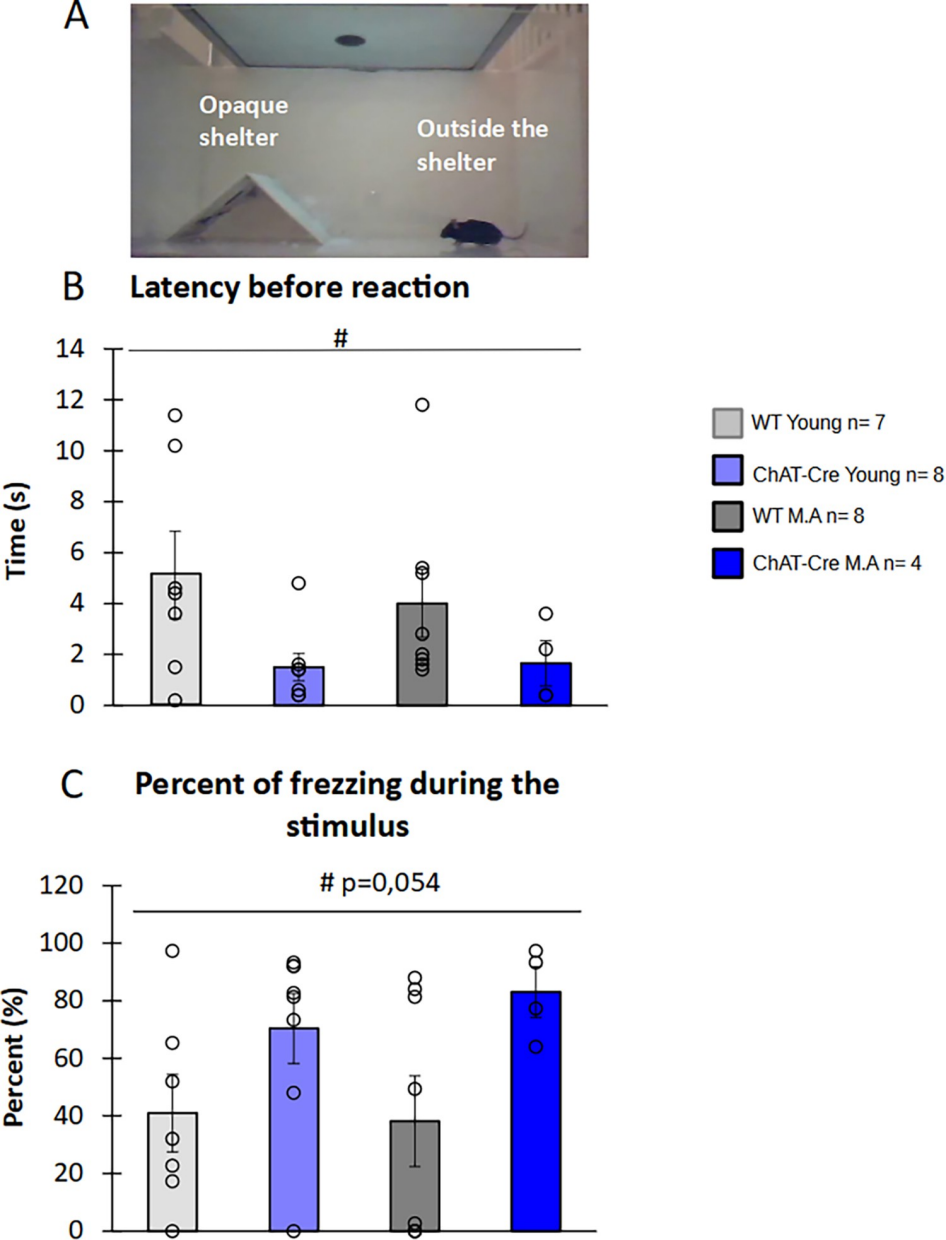
Behavioral response to threatening stimulus. A) Schematic representation the looming experiment. B) Latency of flight or freeze responses. C) Percentage of freezing time. Were only considered mice which were outside of the shelter when the looming stimulus occurred. WT young n = 7, ChAT-Cre young n = 8, WT Middle Age n = 8, ChAT-Cre Middle Age n = 4. Data are presented as means ± SE; *p < 0.05; **p<0.01; ***p<0.001; Kruskal-Wallis rank-sum test analysis then Wilcoxon and d signed-rank tests were applied when appropriate. # represents the genotype effect and ◆ the age effect. Kruskal-Wallis results were not represented for simplicity.

The test allows to evaluate whether mice perceived visual stimuli. A set up of this experiment is shown in Fig 6A. The latency of the first reaction (freeze or flight) after a visual threatening stimulus didn’t significantly differ between groups (H = 6.92, df = 3, p = 0.074, Fig 6B), possibly because of an important variability within the young WT group. However, ChAT-Cre animals responded significantly faster than WT animals (W = 36.5, p <0.01) with no change with age (W = 75.5, NS, Fig 6B). Interestingly, when considering the percentage of freezing during the threatening stimulus, even with no significant difference between groups (H = 5.56, df = 3, NS), ChAT-Cre mice tended to show increased freezing time (W = 169, p = 0.054) with no impact of age (W = 128, NS, Fig 6C). ChAT-Cre mice tended to over-react to threatening visual stimuli.

### 3.4) Olfactory ability

**Fig 7:**
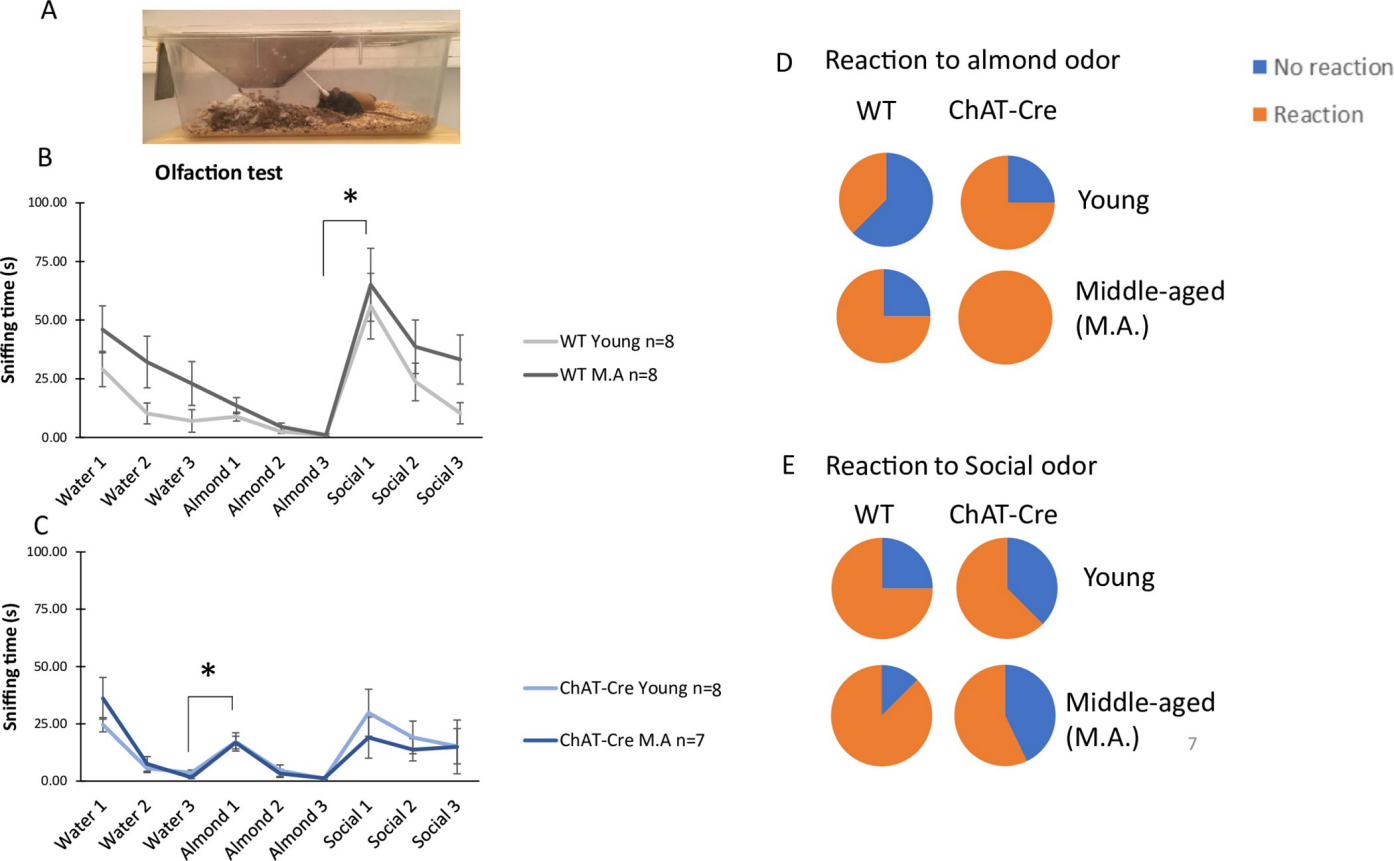
Results of olfaction task. **A)** Picture of the cotton swabs presentation in home cages. B) Time of sniffing cotton swabs with the successive odor presentation of WT mice. C) Time of sniffing cotton swabs with the successive odor presentation of ChAT-Cre mice. D) Percentage mice reacting to almond odor. E) Percentage mice reacting to social odor. WT young n = 7, ChAT-Cre young n = 8, WT Middle Age (M.A.) n = 8, ChAT-Cre Middle Age n = 4. Data are presented as means ± SE; *p < 0.05; **p<0.01; ***p<0.001; Kruskal-Wallis rank-sum test analysis then Wilcoxon and d signed-rank tests were applied when appropriate. # represents the genotype effect and ◆ the age effect. Kruskal-Wallis results were not represented for simplicity.

This test evaluates if animals show olfactory deficits. A set up of this experiment is shown in Fig 7A. After habituation to a novel odor, olfactory abilities were measured by reactions to a novel odor (dishabituation). All groups showed habituation to the neutral water odor by showing a significant decrease in sniffing time during three water presentations (WT young: Friedman chi-squared = 9, df = 2, p <0.05; ChAT-Cre middle-aged: Friedman chi-squared = 13.56, df = 2, p<0.01; ChAT-Cre young: Friedman chi-squared = 11.81, df = 2, p<0.01), except for the middle-aged WTs (Friedman chi-squared = 3, df = 2, NS, Fig 7B and 7C). Only ChAT-Cre mice showed dishabituation during the change from water to almond odor, with a significant increase in sniffing time between the third water presentation and the first almond presentation (young: V = 0, p<0.05; middle-aged V = 3, p<0.05). In contrast, sniffing time of WT animals was not significantly impacted by this change (young; V = 9, NS middle-aged; V = 25, NS).

Habituation to almond odor was globally significant for all groups (WT young: Friedman chi-squared = 15.54, df = 2, p <0.001; WT middle-aged: Friedman chi-squared = 10.75, df = 2, p<0.01; ChAT-Cre young: Friedman chi-squared = 7.75, df = 2, p <0.05; ChAT-Cre middle-aged Friedman chi-squared = 12.07, df = 2, p <0.01, Fig 7B). However, only WT animals showed dishabituation between the last almond odor presentation and the first social odor presentation (young: V = 0, p <0.05; middle-aged: V = 1, p<0.05, Fig 7B). ChAT-Cre mice did not show significant change in sniffing time during this change (young: V = 3, NS; middle-aged: V = 1, NS, Fig 7C).

If WTs habituated to the social odor by showing a significant decrease in sniffing time during the three odor presentations (middle-aged: Friedman chi-squared = 7.71, df = 2, p<0.05; young: Friedman chi-squared = 12.28, df = 2, p<0,01, Fig 7B and 7C), this was not the case for the ChAT-Cre mice (young: Friedman chi-squared = 2.33, df = 2, NS and middle-aged: Friedman chi-squared = 1.18, df = 2, NS). The time of sniffing during the first exposure to the social odor was significantly different between groups (H = 10.685, df = 3, p < 0.05). This results from a significant genotype effect (W = 23, p <0.01) but not from an age effect (W = 86, NS). However, age seems to affect differentially mice of both genotypes: ChAT-Cre young mice didn’t differ from young WTs: W = 8, p = 0.07), but middle-aged ChAT-Cre mice significantly differed from WT middle-aged mice: W = 4, p < 0.05).

It is noticeable that middle-aged WT animals displayed an alteration of water habituation, making dishabituation for the almond odor quite difficult to assess. In order to better apprehend these results, we represented on Fig 7D the percentage of animals showing an increased dishabituation. We clearly see that more than half of young WTs didn’t react to the change towards the almond odor. Similarly, for social odor, the non-significant dishabituation could be explained by the fact that the proportion of animals increasing their sniffing time towards the social odor was reduced in the ChAT-Cre middle-aged group (Fig 7E), where less than 60% of mice reacted to the social odor. The sniffing time of ChAT-Cre mice was significantly different from theoretical proportion (100% of animals reacted to the social odor, Fisher’s Exact Test, p < 0,05). Therefore, we can conclude that ChAT-Cre mice have an olfactory impairement in the detection of social odors.

These results show that WT mice exhibited the expected dishabituation to the social odor but not to the almond odor. In contrast, ChAT-Cre mice showed dishabituation to the almond odor but not to the social one. This confirms that ChAT-Cre mice are able to react to odors but have an alteration in reaction to social odors.

### 3.5) Gene expression

**Fig 8:**
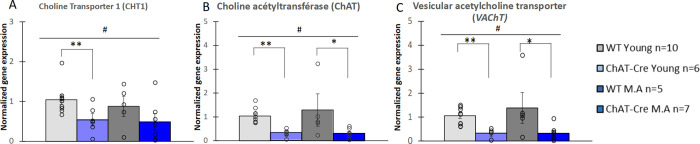
Analysis of *CHT*, *ChAT* and *VAChT* gene expression in the dorsal striatum. Quantification of CHT-mRNAs (A), ChAT-mRNAs (B) and VAChT-mRNAs (C) by RT-qPCR. mRNA levels are presented relative to the young WT group. WT young n = 10, ChAT-Cre young n = 6, WT Middle Age (M.A.) n = 5, ChAT-Cre Middle Age n = 7. Data are presented as means ± SE; *p < 0.05; **p<0.01; ***p<0.001; Kruskal-Wallis rank-sum test analysis then Wilcoxon and d signed-rank tests were applied when appropriate. # represents the genotype effect and ◆ the age effect. Kruskal-Wallis results were not represented for simplicity.

We next investigated the expression of genes required for the cholinergic presynaptic function. CHT-, ChAT- and VAChT-mRNAs were analyzed by RT-qPCR in the dorsal striatum of young and middle-aged mice. A significant difference between groups was found for the expression of these three genes (CHT: H = 8.362, df = 3, p < 0.05; ChAT: H = 17.296, df = 3, p <0.001; VAChT: H = 13.873, df = 3, p <0.01, respectively). This results from a genotype effect (CHT: W = 36, p <0.01; ChAT: W = 19, p <0.001; VAChT: W = 17, p <0.001) but not from an age effect (CHT: W = 120, NS; ChAT: W = 129, NS; VAChT: W = 111, NS, Fig 8). Young and middle-aged ChAT-Cre mice displayed a 50% and 45% decrease of CHT-mRNA levels as compared to WT mice, respectively. This reduction of CHT-mRNAs was significant for young mice (WT young vs ChAT-Cre young: W = 8, p <0.01) but not for middle-aged mice (WT M.A vs ChAT-Cre M.A: W = 9, NS, Fig 8A). The levels of ChAT-mRNAs and VAChT-mRNAs were also lower in ChAT-Cre mice than in WT mice. These genotype-dependent variations were identical for both transcripts (i.e. 70% in young mice and 73% in middle-aged mice) and were significant whatever the age of the mice (Young mice: ChAT, WT vs ChAT-Cre mice: W = 0, p <0.01, VAChT, WT vs ChAT-Cre: W = 0, p <0.01; Middle-aged mice: ChAT, WT vs ChAT-Cre: W = 4, p<0.05, Fig 8B; VAChT, WT vs ChAT-Cre: W = 4, p <0.05, Fig 8C).

Together, these results show a decreased expression of the genes encoding the cholinergic markers CHT, ChAT and VAChT in ChAT-Cre mice compared to WTs.

### 3.6) PCA analysis

**Fig 9:**
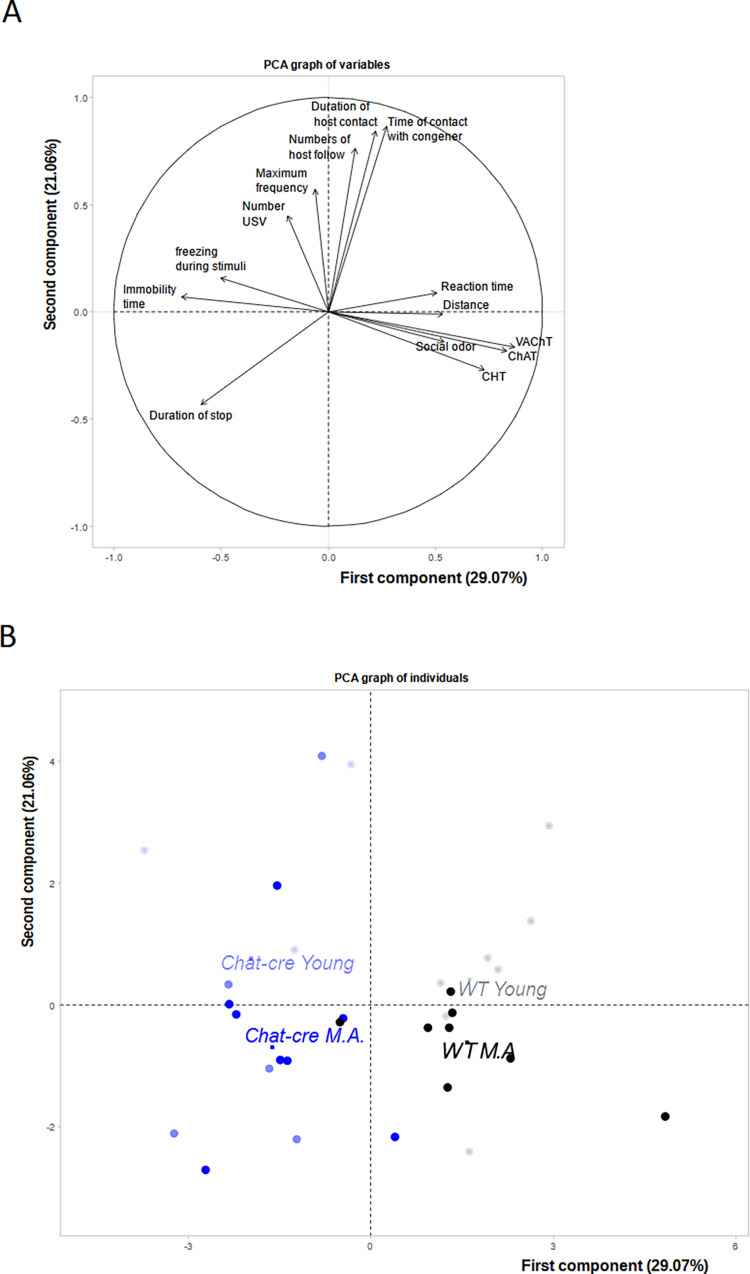
Illustration of principal component analyses performed on behavioral and gene expression parameters. A) Vector representation of the contribution of each parameters. The coordinates of each variable are the correlation coefficients with the two principal components (described in detail in Table 1). Between vectors and between a vector and an axis, there is positive or a negative significant correlation. B) Individual repartition on each individual in variable factor map. The first component explaining about 30% of the data separates WT from ChAT-Cre mice while the second component explaining about 21% of the data separates young mice from middle age mice.

**Fig 10:**
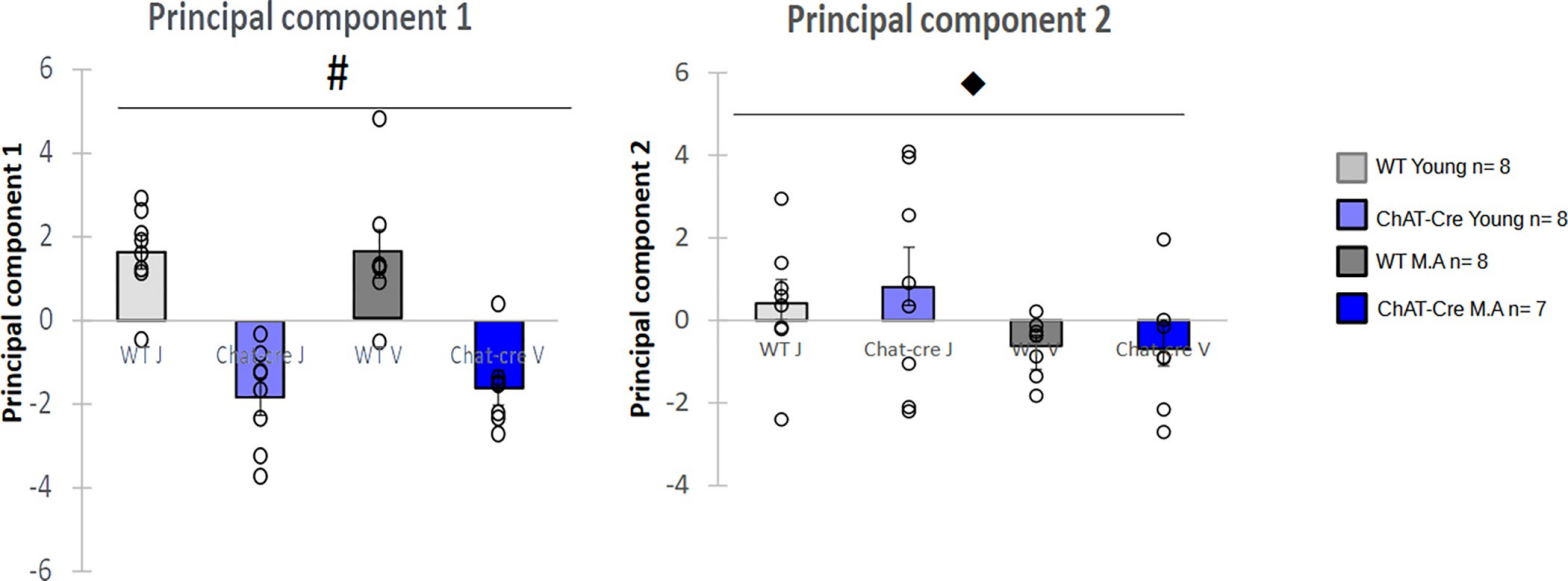
Repartition of each group on the two main components of PCA. A) Repartition of each group on the first main component B) Repartition of each group on the second component. WT young n = 8, ChAT-Cre young n = 8, WT Middle Age (M.A.) n = 8, ChAT-Cre Middle Age n = 7. Data are presented as means ± SE; *p < 0.05; **p<0.01; ***p<0.001; Kruskal-Wallis rank-sum test analysis then Wilcoxon and d signed-rank tests were applied when appropriate. # represents the genotype effect and ◆ the age effect. Kruskal-Wallis results were not represented for simplicity.

We aimed to understand whether there is a link between the behavioral and neurobiological parameters, in the different mouse groups. For this, we carried out principal component analysis (PCA) on the main parameters of each behavioral task: number of follow behavior performed by host mice, number of ultrasonic calls, stop duration, duration of social contact initiated by host mice, time spent interacting with the congener, distance travelled and duration of immobility in the three chamber task, latency to react to a threatening stimulus, freezing proportion during the presentation of the threatening stimulus, sniffing time during almond and social odors, and levels of ChAT, CHT and VAChT gene expression. The variable factor map describing the interaction between these parameters (Fig 9A) shows that two components explain respectively 29.07% and 21.06% of the variance. The description of the components is shown in Table 1. Expression of VAChT, ChAT and CHT in the dorsal striatum, social odor, distance and latency to threat reaction were positively correlated in the first component (PC1). Immobility time, duration of stops and percent of freezzing during the threatening stimulus were negatively correlated to PC1. This component is heterogeneous and seems to be mainly in relation with alterations measured in ChAT-Cre mice. This component allows separating the WT from ChAT-Cre mice. The second component (PC2) is clearly related to dominance and social contact, e.g., number of follow episodes performed by host mice or time of social contact. This component represents age-related social deficit. Importantly, this analysis suggests that cholinergic alterations in the dorsal striatum of ChAT-Cre animals is not related to social behaviors but to aging. PC1 and PC2 were analysed for each group (Fig 10A and 10B). The first component showed a significant group effect (H = 21.13, df = 3, p < 0.01) explained by a genotype effect (W = 4, p <0.001) but not by an age effect (W = 122, NS, Fig 10A). Groups were not significantly different for the second component (H = 5.23, df = 3, NS). Genotype had no significant effect (W = 58, NS, Fig 10B), but there was a significant effect of age (W = 98, p <0.05, Fig 10B).

**Table 1:** Significant results of the PCA performed on the main parameters measured in the behavioral and gene expression experiments. The first component includes cholinergic markers, olfactory reaction to social odor, distance covered in the 3-chamber task and latency to react t a threatening stimulus in the looming task. The second component includes durations of social contact in the SIT, the number of follow episodes and the frequency of USVs. Absence of value indicates no correlation with PCA scores.

	PC1	PC2
**Vacht**	0,87	
**CHAT**	0,83	
**CHT**	0,73	
**Social odor**	0,54	
**Distance**	0,53	
**Reaction time**	0,51	
**Time of contact with congener**		0,86
**Duration of host contact**		0,84
**Numbers of host follow**		0,76
**Maximum frequency**		0,57
**Freezing during stimuli**	-0,50	
**Duration of stop**	-0,59	
**Immobility time**	-0,68	

As illustrated on Fig 9B, the individual repartition of each animal on the two dimensions can discriminate between the four groups of mice. In addition, it shows the distribution within each group and each dimension.

## Discussion

The aim of this study was to determine if ChAT-Cre mice display normal social phenotype and to evaluate their motivation and sensorial abilities that could impact social behavior. We also investigated whether the cholinergic system alterations known to occur with age might impact the progression of age-related cognitive impairments in this mouse line. An additional aim was to extend the characterisation of cholinergic system alterations in this mouse line.

ChAT-Cre young mice exhibit no affiliative or dominance defect. Likewise, they have intact ultrasonic repertoire, suggesting that their communication and emotional state during social interaction are intact. These mice also show normal social motivation in the three chamber tasks, and preserved visual or olfactory sensory abilities. As social interaction was not altered in ChAT-Cre mouse and as striatal cholinergic alteration was not included in the principal component analysis with any social markers, we might suggest that cholinergic innervation toward prefrontal cortex and nucleus accumbens, involved in social interaction and [8,26,68], is likely to be sufficiently functional in ChAT-Cre mice.

Middle-aged animals essentially show decreased dominance in the social interaction task as compared to young ones. Indeed, results of the second component analysis confirm and extend our results showing that affiliative and dominant behaviors along with vocal communication and social motivation are reduced in middle-aged animals. Interestingly, dorsal striatum cholinergic markers deficits seen in ChAT-Cre young mice were not amplified in middle-aged animals compared to young ones. Accordingly, age-related social problems were not amplified in middle age ChAT-Cre animals.

However, we report significant differences between ChAT-Cre and WT mice for the first principal component of the PCA. Using control tasks designed to measure sensory abilities, we observed that ChAT-Cre mice tend to overreact to a threatening visual stimulus, with faster reactions and a tendency to increase freezing behavior as compared to WT mice. In addition, they tend to overreact to a shift in non-social odor (from water to almond) but to react less than WT mice to a social odor (from almond to social). We can also observe a hypolocomotion in three chamber task and increased stop duration in the SIT of ChAT-Cre mice. The first component of PCA shows that this behavioral alteration could be correlated with the decrease of mRNA expression of *ChT*, *ChAT* and *VAChT* genes.

### Reduced levels of CHT-, ChAT- and VAChT-mRNAs in ChAT-Cre mice

Unexpectedly, our results showed a decreased expression of genes encoding essential presynaptic components of cholinergic signaling, i.e. CHT, ChAT and VAChT, in the dorsal striatum of ChAT-Cre mice as compared to WT animals.

One possible explanation for these results would be a bias due to the genetic manipulation *per se* to create the ChAT-Cre mouse line, that would have altered gene transcription efficiency or mRNA stability.

The transcription of these genes is not in theory modified in ChAT-Cre mice, except if the IRES and Cre coding sequence are inserted on a ChAT gene enhancer element, that would be consequently disrupted. Such a genetic modification could induce a decrease of the transcription level of not only the ChAT gene, but also of the VAChT gene, since both ChAT and VAChT genes are intermingled and share one promoter and most probably multiple regulatory sequences [34,35,69]. However, the probability of such an event is low. Moreover, a 50% decrease of either ChAT or VAChT gene expression in heretozygous ChAT or VAChT knock-out mice results in either increased (ChAT+/-) or unchanged (VAChT+/-) levels of CHT-mRNAs [70,71]. Given the fact that CHT-mRNAs are down regulated together with ChAT- and VAChT-mRNAS in ChAT- Cre mice, we do not favor this hypothesis.

The modulation of the expression of these three genes could also occur in part at the post-transcriptional level through a regulatory mechanism of transcript stability involving their 3’ untranslated region (3’UTR). This may be true for ChAT-mRNAs, since the transcription of the ChAT gene in ChAT-Cre mice leads to the synthesis of a « polycistronic » mRNA containing the Cre sequence downstream from the ChAT coding sequence. The 3’UTR of the ChAT-mRNAs is thus modified, which can alter their stability. However, this hypothesis is not valid for VAChT- and CHT-mRNAs, which 3’UTRs remain unchanged in ChAT-Cre mice as compared to WT mice.

Another hypothesis that may explain the concomitant down regulation of the three mRNAs is a potential toxic effect of the Cre recombinase. Previous studies have shown that persistent overexpression of Cre can mediate illegitimate recombination between pseudo-loxP sites within the mouse genome, inducing chromosomal alterations and cytotoxicity [49–51]. In ChAT-Cre mice, Cre expression is under the control of the cholinergic gene locus and is easily detected in adult brain [47]. Since the presence of ChAT- and VAChT-mRNAs was demonstrated from the embryonic stage in WT animals [72–74], Cre is likely present from early development to adulthood in the cholinergic neurons of ChAT-Cre mice. This prolonged chronic expression of Cre might have exerted a toxic effect selectively in cholinergic neurons leading to cellular and molecular alterations including changes in cholinergic gene expression.

It should be noted that studies in different mouse models of cholinergic dysfunction have highlighted the ability of CHT to provide a functional compensation [for review, Bazalakova and Blakely, 2006]. In particular, CHT is upregulated to compensate for hypocholinergic state and sustain WT ACh levels in adult ChAT+/- mice [70] and this compensatory mechanism occurs from the second postnatal week [75]. The fact that CHT gene expression is reduced together with that of ChAT and VAChT genes in ChAT-Cre mice suggests that the mechanism underlying the down regulation of these three genes may affect the number of cholinergic cells rather than the level of expression of these genes within cholinergic cells.

The present study extends a previous characterization of young ChAT-Cre mouse line [47]. In particular, these authors reported that the amount of ChAT protein in the hippocampus is lower than in WT mice, which is consistent with our result on ChAT-mRNAs. However, they found that the level of VAChT protein is comparable to that of WT animals. Theses discrepancy with our results on VAChT-mRNAs may be due to the fact that the two studies were performed on different brain structures, i.e. hippocampus and striatum. In addition, VAChT-mRNA translation could be upregulated in ChAT- Cre mice to compensate for the reduced VAChT-mRNA levels in order to maintain a normal ACh release.

### Age-related changes in ChAT-Cre mice

In WT as in ChAT-Cre mice, PCA analyses evidenced that ageing (11-12-month-old mice) induces a decrease in affiliative and dominant behaviors along with no difference in the vocal communication during social interaction. We can therefore suggest that social motivation was reduced in middle-aged animals. Several previous works described a decrease of social contact with age (20–24 months), either in rats [62] or in mice [76]. However, only a few studies described the evolution of social cognition at middle age, and they reported a decrease of social contact in middle-aged mice (11–12 months old) as compared to young adults (2–3 months old) [63,76]. As a cluster of clues indicates that cholinergic system alterations underlie age-related cognitive impairments [53,55,77,78], it was crucial to know how age impacts ChAT-Cre mice, and whether these changes can occur as early as at middle-age. Interestingly, age effects were not aggravated by genotype on a behavioural point of view. Compared to WT mice, we only observed a marginally significant decrease in time spent in the social area for middle-aged ChAT-Cre mice, with normal time spent in social contact, in the three-chamber test. Similarly, alteration of dorsal striatum cholinergic markers shown in ChAT-Cre mice was not amplified in middle-aged animals compared to young ones. From our results, there is no evidence that ageing accelerates damage to the cholinergic system in ChAT-Cre mice.

### Anxiety-related behavior in ChAT-Cre mouse?

Decreased latency to react to the threatening stimulus might reflect an increased anxiety in ChAT-Cre mice. However, ChAT-Cre young mice didn’t have significative difference in anxiety in open field and elevated plus maze tasks [47]. Our results didn’t show any difference in vocalization between ChAT-Cre and WT mice. USVs are emotional markers in mice and rats [22]. In particular, it was shown that anxious mice display decreased numbers of USV and lower USV mean frequencies (<60kHZ) than non anxious mice [22,79].

The neurobiology of anxiety is extensively described in the literature. The main structures implied in fear and anxiety are the mPFC, the amygdala, the locus coeruleus, the hippocampus and the periaqueductal gray (PAG) [80–83]. ACh plays an important role in anxiety. For example, muscarinic receptors of the ventral hippocampus are involved in the induction of anxiety and their activation promotes social avoidance [84]. In the amygdala, ACh may regulate fear learning and extinction of fear anxiety-like behavior [83]. Moreover, nicotinic receptor activity can trigger a bimodal effect in the brain: low doses of nicotine can have an anxiolytic effect, whereas high doses can have an anxiogenic effect [85].

Together, the decreased latency to react to the threatening stimulus didn’t seem to be triggered by anxiety problems. Future analyses will be necessary to understand this phenomenon.

### Alteration in olfaction in ChAT-Cre mouse?

We measured the sniffing time that mice spend toward the coton swab. We can expect that a mouse which has an efficient olfaction would not necessarily stand close to a stimulus. Both an olfactory problem or an attractive odor could promote sniffing duration. Water and almond were neutral odors [67]. Therefore, these odors may not trigger behavioral approach. Following this hypothesis, we can imagine that young WT mice don’t have any problem to detect non-social odors. However, at middle age a larger proportion of mice (WT and ChAT-Cre alike) react to almond odor (Fig 7D). This can reflect an olfactory problem. Another hypothesis could be that the sniffing time is a very limited measure and that the time spent near the cotton bud could show a difference in olfaction.

Results show that the WT mice react significantly to social odors, suggesting that social odor may be more attractive than a neutral odor such as almond.

ChAT-Cre mice displayed no significant reaction toward the novel social odor. In rodents, the olfactory system contains two separate sensory subsystems, the main olfactory system and the vomeronasal system (VNO) projecting to the accessory olfactory bulb (AOB) [86,87]. The main olfactory system allows to detect all odors (such as water and almond) and VNO detectes pheromones and kairomones present in social odors [88]. Several studies have shown that lesions of VNO alter the perception of social odors (such as that of females or of predators for example) [87,89]. Olfactory bulbs receive dense cholinergic inputs from the basal forebrain and activation or inhibition of this innervation modulates odor detection [90], while the cholinergic tone of the AOB is involved in olfactory processing [90–92]. Electrophysiological experiments demonstrated that muscarinic modulation of olfactory bulbs induces opposite effects on granule cells of AOB and on MOB suggesting a complex cholinergic modulation of non-social and social odors [90]. We might suggest that in ChAT-Cre animals, an alteration of the cholinergic tone leading to differential impact to the main or vomeronasal olfactory system could in part sustain our results.

### Locomotion alteration in ChAT-Cre mice

We show that ChAT-Cre animals travel less distance and spend more time immobile than WT animals in the three-chamber test, thereby confirming previous results [47]. Using the PCA analyses, we found that locomotor parameters belong to the same dimension than cholinergic parameters. Therefore, we can suggest that alteration of cholinergic system triggers locomotion problems.

Howe and collaborators [2019] showed the interplay between ACh and dopamine in the striatum in locomotor processes. ACh might also influence locomotor circuits through pontine cholinergic nuclei of LDTg and PPTg that send cholinergic afferents to the substantia nigra pars compacta (SNpc) and to the ventral tegmental area (VTA), leading to dopaminergic modulation of the striatum [93]. Influence of the cholinergic system on locomotion has been in part dissected [25,93,94]. In particular, using lentiviral vectors to selectively restore cholinergic transmission in the SNpc or the VTA, Avale and collaborators [2008] evidenced the critical role of the cholinergic input of laterodorsal and pedunculopontine tegmental nuclei on the switch between ample locomotion and slow exploration. ChAT-Cre mice showing reduced distance travelled and greater immobility could be more affected in navigation rather than in exploration, suggesting an altered cholinergic modulation of the striato-nigral pathway. In our study, ChAT-Cre mice have intact social motivation and exploration behavior. Regarding the well-known role of dopaminergic and ACh interplay in the nucleus accumbens for reward, motivation and goal directed behavior [68,95–97], we might suggest that the cholinergic influence on the mesolimbic pathway is functional in these mice. In fact, only 20% of projections of LDTg or PPTg on VTA are cholinergic, whereas more than 90% of LDTg or PPTg projection on SNpc are cholinergic [98]. This suggests a greater sensitivity of the nigro-striatal pathway than of the VTA-accumbens pathway to cholinergic disruption.

### Decision alteration

Even if our result show that ChAT-Cre mice have no alteration in organisation of social contact, dominance or number of stop behavior, these mice increased the duration of their stop during social interaction. Our team previously showed that stop behaviors are choice points from which a large panel of behavioral sequences can be chosen during social interaction [29,64,65] and that they are sensitive to monoaminergic-cholinergic prefrontal interaction [29]. It is thus difficult to rule out a possible alteration of decision-making process in Chat-Cre mice in terms of delayed action selection. This interpretation is supported by the fact that disruption of the cholinergic tone in the striatum impairs action selection [8,9,27,28,99–101]. However, our results show that the consequences of such alteration is limited, in the social domain we are looking at.

## Conclusion

The aim of this study was to characterize the social phenotype of ChAT-Cre mice and to evaluate the evolution of their social behavior with age by focusing on mid-life. The results first confirm an alteration of the cholinergic system in ChAT-Cre mice. However, we show that this cholinergic alteration doesn’t disrupt social interaction, except for possible increased decision time. We also identified a subtle sensory deficit, specific to social olfactory detection, associated with a tendency to increase anxiety. ChAT-Cre mice are thus not identical to WT animals. It is thus important to consider the phenotypic differences described in this study and to perform adequate controls in order to rigorously interpret the experimental results obtained with ChAT-Cre mice in the future.

## Supporting information

S1 Data (XLSX)
